# Metallic copper spray – a new control technique to combat invasive container-inhabiting mosquitoes

**DOI:** 10.1186/s13071-015-1180-z

**Published:** 2015-11-09

**Authors:** Norbert Becker, Thin Thin Oo, Nino Schork

**Affiliations:** German Mosquito Control Association (KABS), Institute for Dipterology, Georg-Peter-Süß-Str. 3, 67346 Speyer, Germany; University of Heidelberg, Im Neuenheimer Feld 230, 69120 Heidelberg, Germany

**Keywords:** Metallic copper spray, Invasive mosquitoes, *Ochlerotatus japonicus*, *Aedes albopictus*, Cost-effective, Sustainable mosquito control

## Abstract

**Background:**

The control of container-inhabiting mosquitoes is mainly based on environmental management with special emphasis on community participation e.g. source reduction by elimination or modification of water bodies. However, citizens are often not aware of the problems related to urban mosquito control or just ignore the advice provided during anti-mosquito control campaigns. In particular, cemeteries contain favourite breeding sites for container-inhabiting mosquitoes like *Ochlerotatus j. japonicus*, *Culex pipiens* s.l./*Cx. torrentium*, *Aedes aegypti* or *Aedes albopictus.* In our study, we investigated whether metallic copper e.g. in form of copper spray can be a suitable and cost-effective tool to combat mosquito breeding in vases or other similar small containers where no commonly used insecticides can be applied.

**Methods:**

The effect of metallic copper spray in comparison to 5 Euro cent coins or copper tubes at different dosages and water qualities applied in small water collections such as widely used plastic grave vases were evaluated by assessing the mortality rates of larvae of *Oc.j. japonicus*, *Cx. pipiens* s.l./*Cx. torrentium* and *Ae.aegypti*. Different water qualities were tested to assess the influence of pH on the solubility of the copper ions. The copper concentrations were quantified using ICP/MS (Inductively coupled plasma/Mass spectrometry) in relation to the exposure time and mortality rates of mosquito larvae. All statistical analyses were computed using JMP 10.0.2 (SAS Institute Inc., 2012, Cary, NC, USA).

**Results:**

Dosages of less than 500 ppb of copper in the water of small containers led to a 100 % mortality rate after 2 weeks for all tested mosquito species by using one or more 5 Euro cent coins/vase. When the interior surface of plastic grave vases was covered by metallic copper spray, all of the tested larvae died after 7–10 days in the laboratory and under field conditions the reduction rate was 99.44 % for *Oc.j. japonicus* and 99.6 % for *Culex pipiens* s.l*./Cx. torrentium* larvae for a period of about 3 months.

**Conclusion:**

The use of metallic copper spray provides a sustainable control of container-inhabiting mosquitoes at low costs. The amount of dissolved copper in water (about 500 ppb) is far below the critical value for drinking water according to the WHO recommendations and is therefore not detrimental for the environment.

## Background

During the course of evolution, over hundreds of million years, mosquitoes have survived in many different natural and artificial aquatic habitats, amongst which are small water collections ranging from flower vases, water barrels, buckets, cans, water catch basins, bird baths, tree-holes and many more artificial and natural water bodies [1]. In particular, this kind of breeding site is frequently found in human settlements where container-inhabiting mosquitoes can cause severe nuisance problems and can even be vectors of disease pathogens such as dengue, Chikungunya or West-Nile viruses [1]. The globalisation resulting in increased international trade and human mobility is responsible for the quick spread of pathogens and exotic organisms such as container-inhabiting *Aedes/Ochlerotatus* mosquitoes. Amongst them, the most important are *Ae. (Stegomyia) albopictus* (Skuse 1895), *Ae. (Stegomyia) aegypti* (Linnaeus, 1762), *Oc. (Finlaya) japonicus* (Theobald, 1901), *Oc. (Finlaya) koreicus* (Edwards, 1917), *Oc. (Finlaya) triseriatus* (Say, 1823) and *Oc. (Finlaya) atropalpus* (Coquillett) [2–4, 5]. The worldwide spread of these mosquitoes is based on their specific biological traits: the ability of the larvae to survive desiccation in eggshells for many months or even years, which favours their transport with goods such as used tyres or ornamental plants such as ‘lucky bamboo’ from one continent to another [2]. The plasticity of the genome enables these exotic mosquitoes to adjust to different environmental and ecological conditions. An additional factor supporting the establishment of exotic mosquitoes is the ongoing climate change. Globally rising temperatures along with increasing events of heavy precipitation create far better conditions for both the introduction and establishment of mosquito populations, as well as vector-borne pathogens [6].

In Germany, two exotic mosquitoes are regularly found, namely *Oc. j. japonicus* (the Asian bush mosquito) and *Ae. albopictus* (the Asian tiger mosquito). *Ochlerotatus j. japonicus* occurs widespread in Southwest and Central Germany and can be defined as an established species [7–9]. The climate conditions in its origin of distribution in East Asia (Japan, South-China, Corea, Taiwan and the eastern part of the Russian Federation) are similar to the climate of Central Europe which favoured the establishment of this species in Europe. Major breeding sites are artificial containers like water drums, flower vases, tree-holes, but also rock pools in river beds e.g. in mountainous areas, can also be inhabited. A second exotic species, *Ae. albopictus,* is regularly found in South-Germany [10]. In 2007, an ovitrap located at a rest-area along highway A5 from Italy (via Switzerland) contained eggs of *Ae. albopictus* [10] and in 2012, 2013 and 2014 more than 50 adults were caught in BG sentinel traps aand additional egg batches in ovitraps, amounting to several hundred eggs in the frame of a nationwide surveillance program e.g. at the A5 and A93 [11, 12]. Massive local reproduction in the Upper Rhine Valley has also been observed in the City of Freiburg in 2014 and 2015 (Jöst personal communication, [13]). Adults of *Ae. albopictus* are regularly introduced into Germany by vehicles originating e.g. from Italy where *Ae. albopictus* is abundant and are obviously in the process to establish in South-West Germany. The introduction of *Oc. j. japonicus* is most probably related to the import of goods e.g. used tyres from the U.S.A. or ornamental plants or flower vases from Asia (e.g. China). *Ochlerotatus j. japonicus* was first detected in Central Europe in 2009 [14]. The control of these species is crucial because they are vectors of human pathogens, particularly arboviruses. *Ae. albopictus* is a vector of 22 arboviruses, including Chikungunya, dengue, West Nile, and yellow fever viruses as well as *Dirofilaria immitis* (the dog heartworm) [1, 15, 16]. It is assumed that the Asian tiger mosquito is also involved in the autochthonous transmission of dengue viruses in Southern France and Croatia, as well as in the outbreak of Chikungunya fever in Italy 2007 [17–19]. *Oc. j. japonicus* is a competent vector of several arboviruses, such as West Nile (WN) virus and Japanese encephalitis (JE) virus, but it can also transmit St. Louis encephalitis, Eastern Equine encephalitis and La Crosse virus and is considered a significant public health risk [20–24, 16].

Traditionally, control measures comprise physical, biological and chemical control tools supported by community participation [1]. Physical control measures related to exotic mosquitoes especially include environmental management of mosquito breeding sites with special emphasis on community participation e.g. source reduction by elimination of unnecessary water bodies, by collecting, recycling and disposing of containers and waste; the careful covering of water containers, e.g. with lids or netting to prevent egg laying; to empty, clean and refill drums or vases on a weekly basis; storage under roofs (e.g. used tyres) or discard containers; modify design or repair (e.g. roof gutters, water catch basins, water storage tanks); the filling of water-holes (e.g. potholes) with sand or cement or by draining the water to avoid the collection of water [2].

Biological control measures against container-inhabiting mosquitoes are mainly based on microbial control agents, insect growth regulators or predators like copepods. The application of sterilised *B.t.i.* (Vectobac® DT/Culinex®) tablets can kill container-inhabiting mosquitoes over a period of several weeks [25–27]. Another recent promising development has been reported with a combined formulation of *Bacillus thuringiensis israelensis* (*B.t.i*.) and *Lysinibacillus sphaericus,* (VectoMax®) which also has a residual mosquitocidal effect against container-inhabiting mosquitoes for several weeks.

Chemical control should only be complementary to environmental management or biological control when disease transmission is threatening humans [28]. Besides biological products such as *B.t.i.* formulations, chemical products (IGRs) such as methoprene, diflubenzuron or pyriproxifen could be applied. Space spraying of adulticides should only be applied in case of emergencies [29].

Genetic control of container-inhabiting mosquitoes (e.g. Sterile-Insect-techniques, SIT) has been used in practice against mosquitoes on a number of occasions [30–32], sometimes to explore and validate aspects of the technique, and sometimes to attempt to control mosquito populations.

During the surveillance of activities related to the spread of *Oc. j. japonicus* in Germany, it was observed that mosquito larvae were never found in copper vases in cemeteries. This observation proves the results of Romi [33] and Bellini [34], who found that copper prolonged the development of mosquito larvae and even killed them at a certain copper concentration. In Italy copper vases are frequently used in cemeteries and in Florida, a reduced mosquito production in cemetery was observed where vases with copper liners were used [35]. Copper formulations have been known as effective fungicides for more than 100 years [36, 37]. Copper is also an important trace element for humans and is not toxic for humans at lower dosages. The WHO allows copper concentrations in drinking water up to 2 ppm [38].

In this study, the efficacy of copper with special regard to copper spray has been evaluated to combat container-inhabiting mosquitoes such as *Oc.j. japonicus*, *Cx. pipiens* s.l. (Linnaeus 1758), *Cx. torrentium* (Martini 1925) and *Ae. aegypti* in flower vases or comparable small containers in order to limit the distribution of exotic mosquitoes.

## Methods

In a series of experiments, the effect of copper and other metals on the development of container-inhabiting mosquitoes such as *Oc.j.japonicus*, *Cx. pipiens* s.l./*Cx. torrentium* (the two species were not distinguished in the larval stage) and *Ae. aegypti* have been investigated. In particular, the effect of copper spray (Kupfer-Lack-Spray, Fritz-International Limited, Kirchberg, Germany) as a cost-effective tool in comparison to the use of 5 Euro cent coins (weight: 3.9 ± 0.1 g; surface: 8.3 cm^2^) and copper tubes (weight: 8.26 ± 0.8 g; surface: 19.43 cm^2^) applied in plastic grave vases (volume: 750 ml) regularely used in Germany was evaluated.

### Series 1: Assessment of the efficacy of different metals against the developmental stages of container-inhabiting mosquitoes

Five copper, steel, zinc and glass cups were filled with 38 ml of tap water (pH: 7.2; conductivity: 70 μS) and then 20 second/third instars of either *Cx. pipiens* s.l./*Cx. torrentium* or *Ae. aegypti* were added in 2 ml of water to the test vessels. A small amount of Tetra-Tabimin (a tip of a spatula) served as a food resource. The vessels were randomly assorted. The mortality readings were conducted after 1, 2, 3, 5, 7, 9, 10 and 13 days.

### Series 2: Evaluation of the sensibility of various mosquito species to copper

Thirteen green plastic grave vases were each filled with 750 ml of tap water (pH: 7.2; conductivity: 70 μS). In each of fifteen vases a 5 Euro cent coin was added. The same number of vases remained untreated as control. In each of five vases with copper coins either 20 second/third instars of *Oc. j. japonicus* or *Cx. pipiens* s.l./*Cx. torrentium* or *Ae. aegypti* were added including a small amount of Tetra-Tabimin powder as food resource. In each of five untreated vases the same number of larvae of the tested species were added as control. The test vessels were randomly assorted and the mortality readings were conducted at 1, 2, 3, 5, 7, 10, 13 and 15 days.

### Series 3: Assessment of the influence of various copper concentrations on the mortality of the container-inhabiting species *Aedes aegypti* as a model species

Larvae of a *Aedes aegypti* colony were used to assess the influence of various copper concentrations on the mortality rate. Twenty vases were filled with 750 ml tap water (pH: 7.2; conductivity: 70 μS) and 20 second/third instars of *Ae. aegypti* as well as a small amount of TetraTabimin were added to each vase. Each of five vases were treated with either one 5 Euro cent coin (weight: 3.9 ± 0.1 g; surface: 8.3 cm^2^) or four 5 Euro cent coins or one copper tube (length: 3 cm, diameter: 2 cm, weight: 7 g). Five vases were left untreated as control. The mortality readings in the randomly assorted vessels were done 1, 2, 3, 4, 6, 7, 10, 12 and 13 days after treatment.

### Series 4: Assessment of the effect of different water qualities with special regard to the pH on the solubility of copper

Forty grave vases were filled with 750 ml of water of different quality: 20 vases with water from a well out of a sandstone mountain area (Heidelberg, Germany) with a pH of 4.4, conductivity of 90 μS, and another 20 vases were filled with tap water (pH: 7.2; conductivity: 70 μS). Five vases of each water quality were treated with one 5 Euro cent coin and then 20 second/third instars of either *Ae. aegypti* or *Cx pipiens* s.l./*Cx. torrentium* were added, including small amounts of TetraTabimin. Five vases served as a control without coins for each water quality and mosquito species. The mortality readings in the randomly assorted vessels were done 1, 2, 3, 5, 7, 10, 12, 13 and 15 days after treatment.

### Series 5: Assessment of the efficacy of copper spray in the laboratory

The inner surfaces of four grave vases were uniformly sprayed with a commercially available copper spray (Kupfer-Lack-Spray). The spraytime/vase was about 10 s, the amount of copper spray/vase ca. 2 g. Although the spray dried quickly, the vases were stored for 24 h at room temperature to allow complete drying of the spray. Then, the vases were washed with tap water and each filled with 750 ml of tap water (pH: 7,2; conductivity: 90 μS). In each vase 20 second/third instars of *Ae. aegypti* were added with some TetraTabimin as food resource. Four untreated vases containing the same number of larvae and food served as control. The mortality was determined after 1, 3, 5, 7, 10 and 12 days (Copper spray I). After two weeks, the vases were emptied and kept dry for one week. Then, the test was repeated again three times (Copper spray II, III and IV) with the same vases to assess the long-term effects of the copper spray with intermittent dry phases. The vessels were always randomly assorted.

### Series 6: Determination of the copper concentrations in relation to the exposure time and mortality of *Ae. aegypti* larvae as a model species

Forty-five grave vases were filled with 750 ml of tap water (pH: 7,2; conductivity; 70 μS) and 20 second/third instars of *Ae. aegypti* and some TetraTabimin as food resource were added to each vase. The vases were placed in three rows, each containing 15 vases. In each vessel of the first row, a copper tube (length: 3 cm; diameter; 2 cm; weight: 8.26 ± 0.8 g; surface: 19.43 cm^2^) was placed, and in each vessel of the second row a 5 Euro cent coin was deposited. The third row was kept as a control without copper. On day 0 and after 3, 7, 14 and 18 days, three vases were checked from each test row for the mortality rate and the water of each single vase was used to determine the concentration of copper ions in the water. Copper concentrations in water samples were quantified using ICP/MS (Inductively coupled plasma/Mass spectrometry; Perkin-Elmer, Elan 6000). The calibration of the instrument was done using at least five different concentration levels. The limit of detection was lower than 1 μg/L. All samples were analysed using Copper isotope 63 [39]. The copper concentrations are given as mean values for each of three water samples per sampling day and row including the standard deviation.

### Series 7: Evaluation of the long-term effect of copper spray used in standard grave vases under field conditions

The inner surfaces of 40 grave vases were uniformly sprayed with copper spray according to test series no. 5 (Kupfer-Lack-Spray; spray time/vase: 10 s, amount of copper spray/vase: 2 g). After treatment, the vases were stored for 24 h at room temperature to allow complete drying of the spray. Then, the vases were washed with tap water. Additionally, 40 vases were kept untreated. In each vase, four 3 mm holes were drilled at the 750 ml mark to avoid running off of water after rainfall. The vases were deposit at the 4 boundaries of the cemetery of Leinfelden (close to Stuttgart) which is known to be heavily infested by *Oc. j. japonicus*. At each boundary, 10 treated and 10 untreated vases were grouped in pairs alternating with an untreated and treated vase at a distance of 5 m under bushes. Each of the vases was filled with 500 ml of hay infusion (50 l of rainwater and 500 g of hay were fermented for 3 days and sieved) and 200 ml of rainwater. Every 14 days, the content of each of the vases was poured into a white container and the developing stages were removed and carried to the laboratory for species determination and counting. The water of each individual vase was refilled again, and rainwater was added to achieve a volume of 700 ml. Then, the vases were brought to their previous position. The experiment started in July and finished in September, 2014.

#### Data analysis

Differences in mortality rates among treatments were tested with a univariate survival analysis. Data have been right-censored when an experiment ends without 100 % mortality and some individuals survived. Differences between treatments or species, respectively, were tested by Chi-square approximations with Log-Rank and WilcoxonTests. *P*-values <0.05 indicate significant differences between treatments or species, respectively. Differences in mean abundance between treatments and the effect of exposure time in test no. 7 were tested using repeated measures MANOVA. Since the sphericity-test was significant, an epsilon adjusted Geisser and Greenhouse G-G-tests was applied. To fit assumptions for MANOVA, data were log10-transformed. All analyses were computed using JMP 10.0.2 (SAS Institute Inc., 2012, Cary, NC, USA).

## Results

### Assessment of the efficacy of different metals against the developmental stages of container-inhabiting mosquitoes

**Fig. 1:**
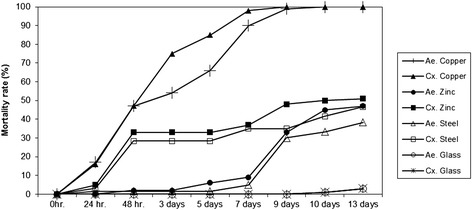
Effect of copper, zinc, steel and glass breeding vessels on the development of *Ae. aegypti* and *Cx. pipiens* s.l*.*/*Cx. torrentium* larvae

All larvae of *Cx pipiens* sl./*Cx. torrentium* and *Ae. aegypti* larvae in the copper vessels were dead after 10 days (Fig. 1). In the same time period, only 45 % and 50 % of *Ae.aegypti* and *Cx, pipiens* s.l./*Cx. torrentium* larvae died in the zinc or 33.3 % and 41.6 % in the steel vessels, respectively. The mortality rate in the glass vessels was 1 % for both species. In summary, the application of copper increased the mortality of *Aedes* sp. and *Culex* spp. significantly more than glass, zinc, or steel (*p* < 0.0001, DF =2). The sensitivity of *Aedes* and *Culex* larvae to copper was more or less the same (*p* > 0.05).

### Evaluation of the sensibility of various mosquito species to copper

**Fig. 2:**
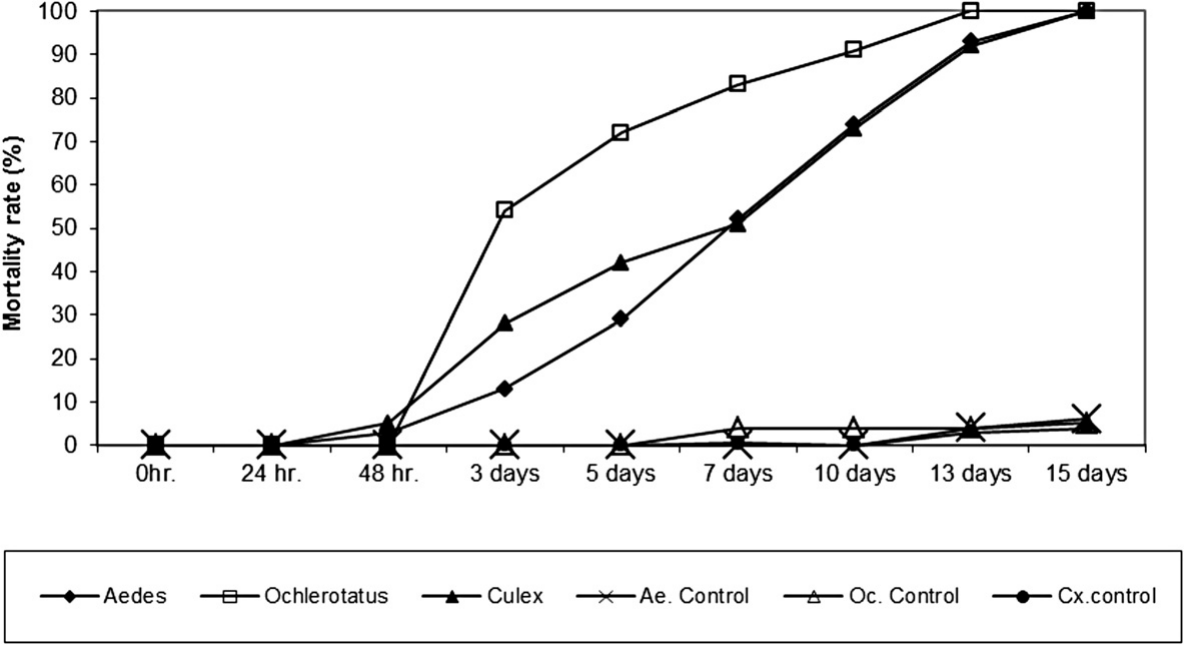
Comparison of the sensitivity of *Oc. j. japonicus*, *Cx. pipiens* s.l*.*/*Cx. torrentium* and *Ae. aegypti* larvae after exposure to 5 Euro cent coins in the breeding containers

In this test series, it could be shown that larvae of *Oc. j. japonicus* are significantly more sensible for copper ions than larvae of *Cx. pipiens*s.l*./Cx. torrentium* and *Ae. aegypti* (*p* = 0.02, DF = 1). After 3 days of exposure the mortality rate was 54 % of *Oc. j. japonicus* larvae, but only 28 % of *Cx. pipiens* s.l./*Cx. torrentium* and 13 % of *Ae. aegypti* larvae. However, after 15 days of exposure, the mortality rates of all species were 100 % (Fig. 2).

### Assessment of the influence of various copper concentrations on the mortality of the container-inhabiting species *Aedes aegypti* as a model species

**Fig. 3:**
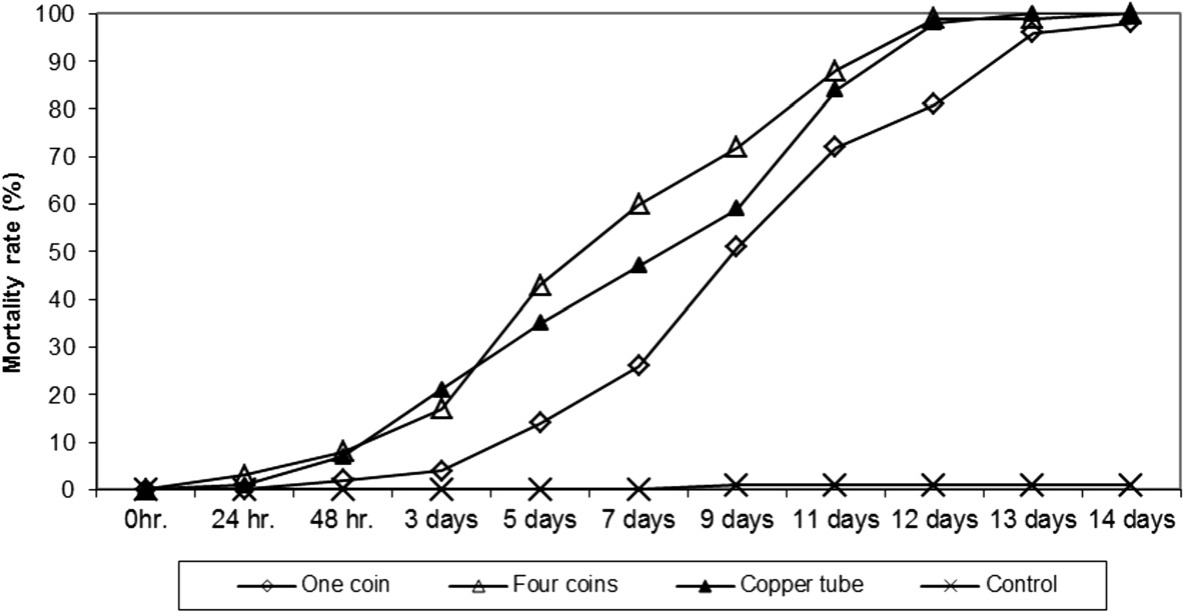
Effect of 5 Euro cent coins in different dosages (1 coin/vase and 4 coins/vase) and copper tubes on the development of *Ae. aegypti* larvae

In this test series, the mortality rates in vases with 4 pieces of 5 Euro cent coins and copper tubes were constantly higher than in the vases with a single 5 Euro cent coin (*p* = 0,035; DF = 1). After 12 days, the mortality rate in vases with 4 pieces of 5 Euro cent coins and copper tubes was 98 % and with a single 5 Euro cent coin only 93 % (Fig. 3). After 14 days, the mortality rate was 100 % in vases with 4 pieces of 5 Euro cent coins or copper tubes, but only 98 % in vases with one 5 Euro cent. In the control vases, the mortality was 1–2 % (*p* < 0.0001; Df = 2).

### Assessment of the effect of different water qualities with special regard to the pH on the solubility of copper

**Fig. 4:**
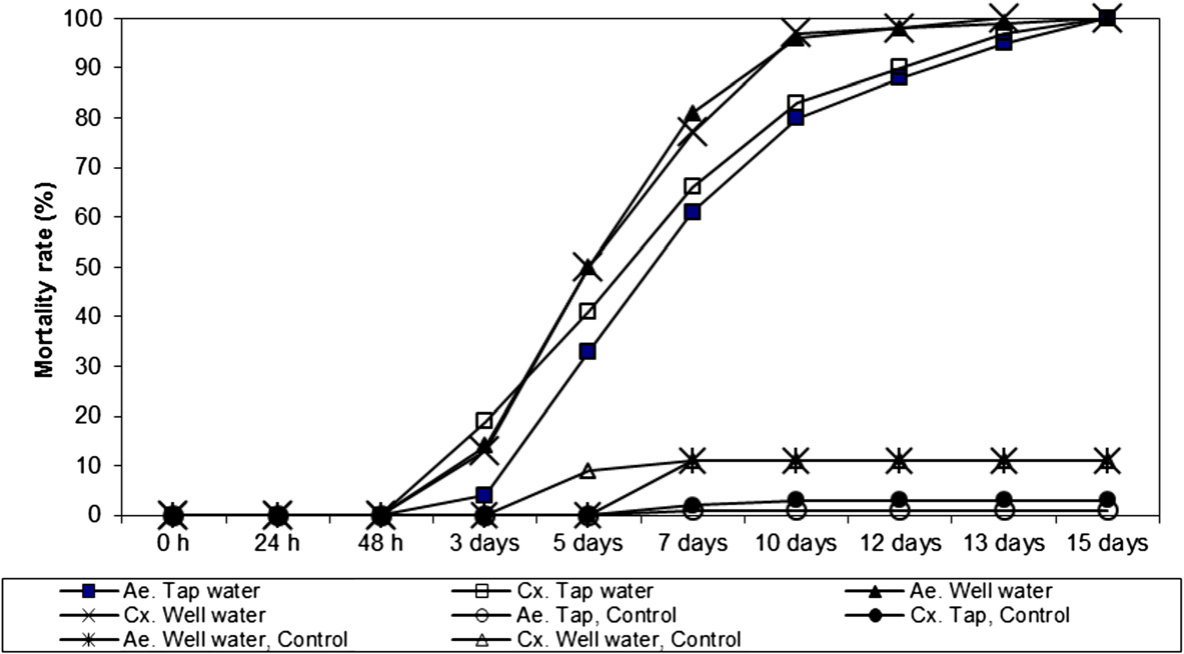
Effect of water with different pH on the solubility of copper and mortality on *Ae. aegypti* and *Cx. pipiens* s.l*.*/*Cx. torrentium* larvae

The mortality rates from day 5 onwards were higher in the test series with water from the well (pH: 4.4) for both mosquito species compared to the trials with tap water. On day 10, the mortality rates in tap water trials were 80 (*Aedes* larvae) and 83 % (*Culex* larvae) compared to 96 % and 97 % in well water; however, after 15 days, all larvae were dead in all test vessels with copper (Fig. 4). The mortality rate in the control vessels never exceeded more than 11 %. However, the results showed no significant differences between treatments (*p* > 0.05, DF =1). Also, no significant differences between species occurred (*p* > 0.05, DF = 1).

### Assessment of the efficacy of copper spray in the laboratory

**Fig. 5:**
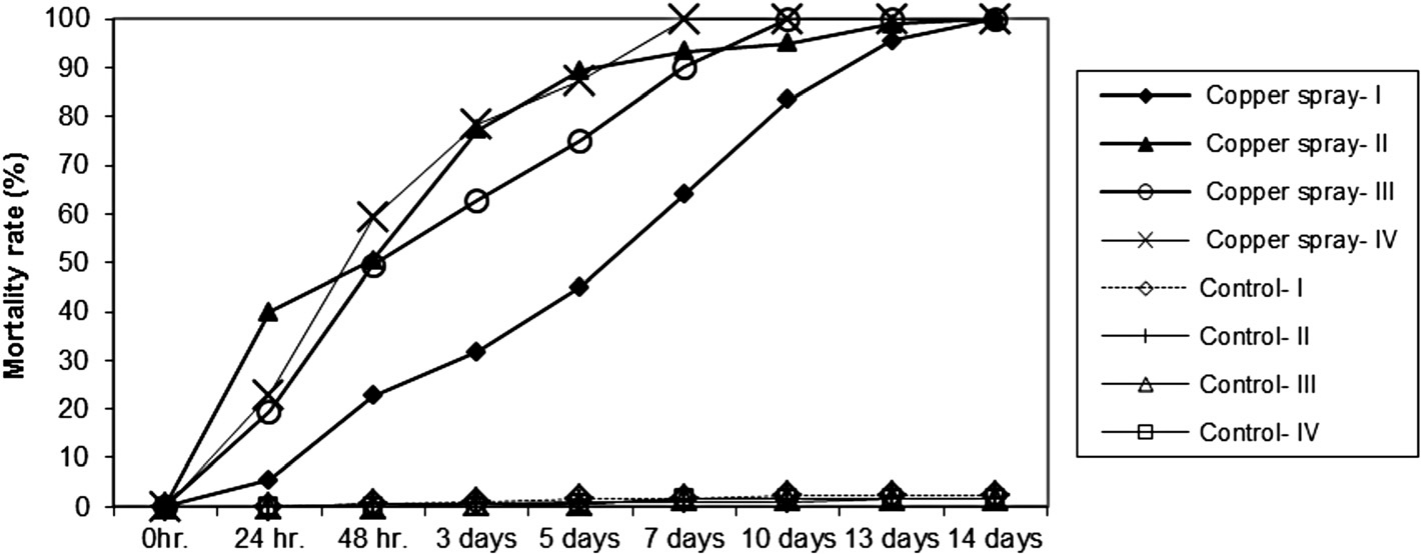
Effect of copper spray on the development on *Aedes aegypti* larvae after three repetitions of intermittent drying out (Copper Spray II-IV) and refilling with water

The mortality of *Aedes* larvae increased significantly after treatments with copper spray compared to controls (*p* < 0.0001; DF = 7). Within 7 to 10 days, all larvae died in the vases treated with copper spray (Fig. 5). The effect of copper spray was increased with the increasing number of refilling of the vases. After the fourth refilling of the vases with tap water, the mortality rate was significantly higher than during the first round of assessment (= copper spray I; *p* < 0.002; DF =3). The time to kill all larvae was half of that needed during the first round of testing, namely 7 days to achieve 100 % mortality compared to 14 days during the first round.

### Determination of the copper concentrations in relation to the exposure time and mortality of *Ae. aegypti* larvae as a model species

In this test series, the concentration of copper in vessels with copper tubes and 5 Euro cent coins in relation to the mortality rate of *Ae. aegypti* larvae was measured. Untreated vases served as a control. The copper concentration in untreated vases was 12.1 ppb (±1.22 ppb) on day ‘0’, 11 ppb ((±0 ppb) on day 3, 1.5 ppb ((±1.5 ppb) on day 7 and 0 ppb at day 14 and 18. The copper dosages in the vases with 5 Euro cent coins were 29.4 ppb ((±1.41 ppb), 74.4 ppb (±9,25 ppb), 18.6 ppb (±5.7 ppb), 18.4 ppb (±1.87 ppb) and 35.6 ppb (±9.00 ppb) at days 0, 3, 7, 14 and 18, respectively. The mortality rates were 0 % on days 0 and 3, 23.3 % (±4.7 %) on day 7, 60 % (±8.2 %) on day14 and 83.3 % (±12.5 %) on day 18. The highest copper concentrations and mortality rates were achieved with copper tubes. The copper concentrations were 37.3 ppb (±5.9 ppb), 360.3 ppb (±47.39 ppb), 329.3 ppb (±45.1 ppb), 309.8 (±55.6 ppb), and 478 ppb (±135.1 ppb) on days 0, 3, 7, 14 and 18, respectively. The mortality rates were 0 % on day 0 and 3, 66.6 % (±4.7 %) on day 7, 80 % (±0 %) on day 14 and 100 % (±0 %) on day 18.

### Evaluation of the long-term effect of copper spray used in standard grave vases under field conditions

The results of this test series demonstrate the high efficacy of copper spray to prevent breeding of *Oc.j. japonicus* and *Cx. pipiens*s.l./*Cx. torrentium* under field conditions for almost three months. During the observation period of 10 weeks, in the untreated vases a total of 1886 and 2612 early larval instars (L. 1&2) of *Oc. j. japonicus* and *Cx. pipiens* s.l. */Cx. torrentium* were counted, respectively. In the treated vases, 1567 and 1219 early larval instars (L. 1&2) of *Oc.j. japonicus* and *Cx. pipiens* s.l.*/Cx. torrentium* occurred during the test period, respectively. The number of early instars of *Oc. j. japonicus* was almost the same in the treated and untreated vases. However, there was a significant difference in the number of early *Culex* larvae between treated (total of 1219 larvae) and untreated vases (total of 2612 larvae).

**Fig. 6:**
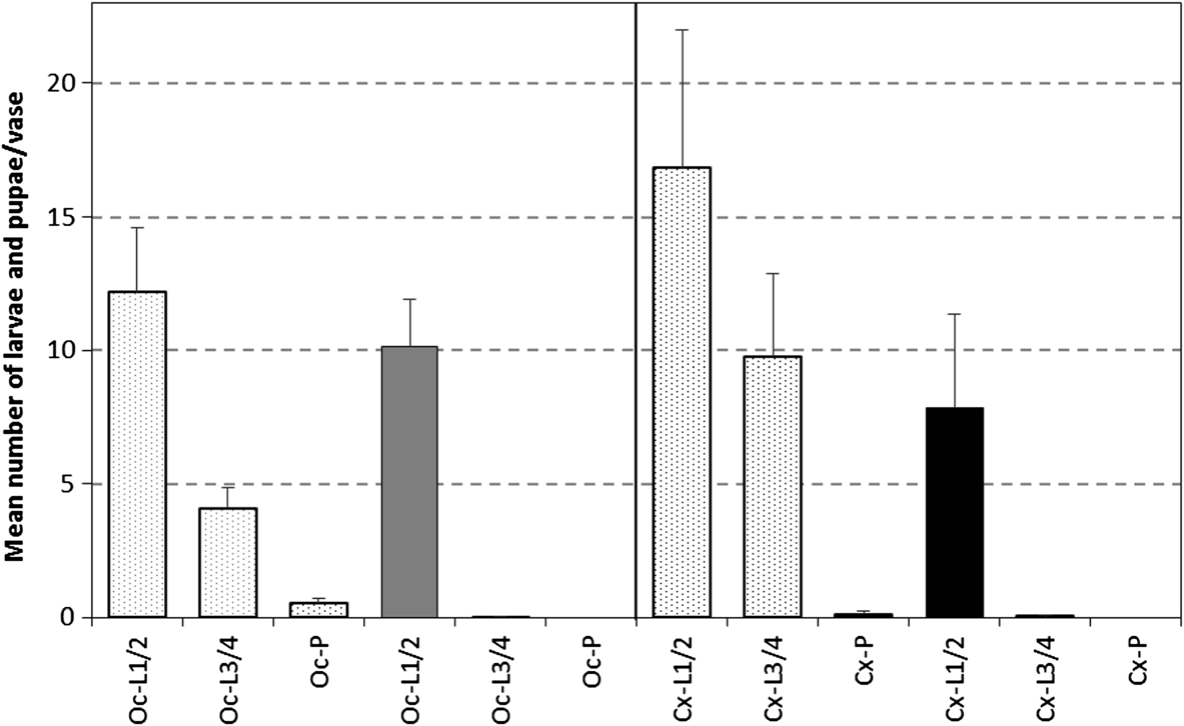
Mean numbers of larvae and pupae/vase of *Ochlerotatus j. japonicus* and *Culex pipiens* s.l*./Cx. torrentium* after and without the application of copper-spray under field conditions during a period of 76 days. *Left side* = *Ochlerotatus* (Oc), dotted = vases without copper spray (plastic), grey = vases with copper spray; *right side* = *Culex* (Cx), dotted = vases without copper spray (plastic); black = vases with copper spray; L1/2 = larval stages 1 & 2, L3/4 = larval stages 3 & 4, P = pupae; bars = standard error, n = 156

The comparison of the number of late larval instars (L. 3&4) and pupae between untreated and treated vases highlights the efficacy of the copper spray treatments. In the untreated vases, a total of 633 late larval instars and 82 pupae of *Oc. j. japonicus,* as well as 1512 late larval instars (L. 3&4) and 3 pupae of *Cx. pipiens/Cx. torrentium,* were counted. On the contrary, in the treated vases, only four late instar larvae of *Oc. j. japonicus* and 6 late larval instars of *Culex*s.l. occurred. Mean abundances of late larval instars and pupae of both species were significantly lower in treated vases (MANOVA: L 3&4: F =57.93, *p* < 0.0001; pupae: F = 23.25, *p* > 0.0001; Fig. 6).

Exposure time showed a significant positive interaction on species in case of all life stages (repeated measures MANOVA with univariate G-G-test: L 1&2: F = 31.27, *p* < 0.0001; L 3&4: F = 7.69, *p* < 0.0001; pupae: F = 7.08, *p* = 0.0013) and also on treatment - however only in case of late larval instars and pupae (repeated measures MANOVA with univariate G-G-test: L 3&4: F = 2.0, *p* = 0.04; pupae: F = 5.52, *p* = 0.0053).

The mortality rate was more than 99 % for larvae of all genera over a period of almost three months.

## Discussion

Human settlements provide ideal conditions for the proliferation of container-inhabiting mosquitoes. In particular, cemeteries are favourite breeding grounds because mosquitoes usually find numerous small containers like flower vases, human visitors as a source of a blood meal, flowers for nectar feeding and bushes as resting places. Therefore, in Germany not only indigenous mosquito species like *Cx.pipiens* s.l./*Cx. torrentium*, *Oc. geniculatus*, *Anopheles plumbeus* and *Culiseta. annulata* are regularly found in such habitats, but also exotic species like *Oc. j. japonicus* and increasingly *Ae. albopictus* in South Germany [11]. In the “German Mosquito Control Association” (KABS) the control of urban mosquitoes is mainly carried out by inhabitants [40]. To assist the population with this, KABS provides information on the biology of container-inhabiting mosquitoes and appropriate control measures e.g. in special flyers and press releases. Culinex®-tablets, which contain toxins of *Bacillus thuringiensis israelensis* (strain AM 65-52, serotype H-14) have been particularly successfully used for more than two decades in this program. The *B.t.i.-*tablets kill mosquito larvae in rainwater containers over a period of several weeks [41]. However, in an environmental sanitation program, the inhabitants are first asked to eliminate unnecessary containers like water-filled buckets or unused flower vases or to cover larger containers with lids, so that mosquitoes are unable to lay their eggs. These measures are difficult to maintain in cemeteries where people use flower vases for religious purposes. In some parts of Germany, the exotic mosquito *Oc. j. japonicus* and increasingly *Ae. albopictus* occur frequently in cemeteries where they multiply during the summer in flower grave vases [8]. Here, control measures have to be sustainable. In our study, it was proven that metallic copper spray shows a long-term killing effect in all of the tested mosquito species. The toxic effect of metallic copper on aquatic organisms has also been documented by Kosalwat and Knight [42], as well as Clements [43]. Furthermore, the negative effect of copper on the development of mosquitoes was documented by O’Meara et al. [35], Bellini et al. [34] and Romi et al. [33]. O’Meara et al. [35] found that significantly fewer vases with copper liners were positive for mosquitoes compared to vases lacking liners or with aluminium liners in cemeteries in Florida.

Bellini et al. [34] studied the effect of copper multiwire electric cable (2 g/l and 8 g/l) on the development of *Ae. albopictus* in the laboratory. Copper concentrations of 424 μg/l and 565 μg/l prolonged the larval development and led to larval mortality, especially in earlier instars. Romi et al. [33] found that a dose of 20 g/l of a multiwire electric cable inhibited the development of *Ae. albopictus*. Copper ion concentration <500 ppb only slightly affected larval development; however, copper concentrations > 1000 ppb inhibited the larval development completely. In field trials, a dose of 20 g of multiwire cable/litre reduced the number of larvae in treated plots by 90 % and a dose of 40 g copper/litre prevented breeding. In our experiments, the copper concentrations were much lower corresponding to 5.3 g/l (5 Euro cent coin/vase) and 11 g/l (1 copper tube/vase). The highest ion concentration was 478 ppb when copper tubes were used. A 5 Euro cent coin in a plastic grave vase was sufficient to kill all larvae of the tested species. With an increasing number of coins per vase, a 100 % mortality rate could be achieved slightly faster. A first field test on a cemetery with a one 5 Euro cent coin/vase was not successful because people dumped the coins by replacing the water and the flowers in the vases. A simple, cost-effective and practical solution could be the use of metallic copper spray to cover the interior surface of plastic grave vases which are extensively used in Germany. A copper layer of less than 2 g/vases which can be sprayed in 10 s is enough to kill the larvae for several months and achieve the same effect as with coins, even when the water was changed three times (Fig. 5). The copper spray technique is cheap, efficient and can be utilised to avoid the colonisation of cemeteries by container-inhabiting mosquitoes, with special emphasis on *Oc. j. japonicus, Ae. albopictus* and *Cx, pipiens* s.l./*Cx. torrentium*. The pH of the water has an effect on the solubility of copper ions. In the experiments with well water at a pH of 4.4, the mortality rate increased more rapidly than in vases with alkaline water; however, the mortality rates achieved after 15 days with both water qualities were 100 % (Fig. 4). It appears that the water quality of a certain area will not significantly influence the efficacy of this technique in routine treatments. An interesting observation in our laboratory was that our mosquito colonies were wiped out when we used water from a certain water source in which copper pipes were used.

The use of copper spray seems to be an appropriate and cost-effective tool to combat container-inhabiting mosquitoes in small breeding sites like flower vases in cemeteries. Uniform coverage of the inner surface of vases or related containers guarantees killing of all mosquito larvae for more than 10 weeks. The costs per treatment are less than 2 Euro cent. The amount of dissolved copper in water is far below the critical value according to the WHO recommendations [38].

The toxic effect of copper on bacteria, fungi and algae has been known for centuries and utilised as fungicides and to inhibit algal growth. It can be assumed that the algicidal and bacterial effect of copper could also have a negative impact on the development of mosquito larvae because of the destruction of the microbiota in the gut of the mosquito larvae. At high dosages (10 ppm) in drinking water, it can be detrimental for humans and can, for instance, damage the liver [44]. This is the reason why copper pipes for drinking water have been replaced with inert materials. The maximum level of copper in drinking water should not exceed 2 ppm according to the German Drinking water regulation (TVO).

The copper dosages used in our experiments were well below the limit allowed for drinking water. On the other hand, copper is a very important trace element for human health. A balanced copper concentration in food is essential for bone formation, for the cardiovascular system and for the function of the central nervous system, as well as to stimulate enzyme activities. The toxic effect on mosquitoes might be caused by the bioaccumulation in larvae and the effect on the microbials in the gut of larvae which are essential for the growth of the mosquito larvae. The accumulation of copper in the larvae has also been proven by a reduction of the copper concentration in the test vessels by increasing the exposure time (see test series 6).

## Conclusion

The use of metallic copper as a naturally occurring compound with its insecticidal abilities is a suitable and cost-effective tool to combat mosquitoes breeding in vases or other similar small containers. In our study, we showed that the use of metallic copper spray provides a sustainable control of container-inhabiting mosquitoes such as *Ochlerotatus j. japonicus*, *Aedes aegypti* and *Culex pipiens* s.l./*Cx. torrentium* at low costs. With one single treatment of the interior wall of a plastic vase a long-term control effect of at least three months can be achieved without the risk of dumping copper pieces, for example when copper coins are lost during the water change. A solid coverage of the interior wall of a vase with copper spray takes only a few seconds and costs about 2 Euro cents. The amount of dissolved copper in water (about 500 ppb) is far below the critical value for drinking water according to the WHO recommendations and is therefore not detrimental for the environment.
